# Molecular methods to detect *Spodoptera frugiperda* in Ghana, and implications for monitoring the spread of invasive species in developing countries

**DOI:** 10.1038/s41598-017-04238-y

**Published:** 2017-06-22

**Authors:** Matthew J. W. Cock, Patrick K. Beseh, Alan G. Buddie, Giovanni Cafá, Jayne Crozier

**Affiliations:** 1 CABI, Bakeham Lane, Egham, TW20 9TY UK; 2 MOFA-PPRSD, P.O. Box M37, Accra, Ghana

## Abstract

*Spodoptera frugiperda* (J. E. Smith) (Lepidoptera: Noctuidae) is a polyphagous pest indigenous throughout the Americas, which recently appeared in Africa, first reported from São Tomé, Nigeria, Bénin and Togo in 2016, and which we now report from Ghana. This species is recognised to comprise two morphologically identical but genetically distinct strains or species in the Americas, and we found both to be present in Ghana. We discuss possible routes of entry to Africa, of which the likeliest is adults and/or egg masses transported on direct commercial flights between the Americas and West Africa, followed by dispersal by adult flight within Africa. Identification of Lepidoptera is normally based on the markings and morphology of adults, and not on the larvae which actually cause the damage, and therefore larvae have to be reared through to adult for authoritative identification. We confirmed that the use of DNA barcoding allowed unequivocal identification of this new pest from Ghana based on the larvae alone. As authenticated barcodes for vouchered specimens of more pests become available, this approach has the potential to become a valuable in-country tool to support national capability in rapid and reliable pest diagnosis and identification.

## Introduction

*Spodoptera frugiperda* (J. E. Smith) (Lepidoptera: Noctuidae) is a polyphagous pest indigenous throughout the Americas^1, 2^, which has recently appeared in Africa^3^, and we have now detected in Ghana. It is regularly intercepted in intercontinental trade^4^ but has not previously become established outside the Americas (a report of its presence in Israel^5^ was based on a misidentification)^2^. The caterpillars of this moth feed on leaves, stems and reproductive parts of more than 100 plant species^6^, causing major damage to economically important cultivated grasses such as maize, rice, sorghum and sugarcane as well as other crops including cabbage, beet, peanut, soybean, alfalfa, onion, cotton, pasture grasses, millet, tomato, potato and cotton^4, 6^. Although widely agreed to be one of the most damaging crop pests in the Americas^7^, economic assessments of crop losses and the costs of control are not comprehensive. However, in Brazil, for example, *S*. *frugiperda* is considered the major insect pest for maize, causing up to 34% reduction in grain yield^8^ and annual losses of US$400 million^9^.

For about 30 years, it has been known that *S*. *frugiperda* occurred in two races, a ‘rice strain’ (R strain) and a ‘corn strain’ (C strain)^10^; the former is thought to preferentially feed on rice and various pasture grasses and the latter on maize, cotton and sorghum, although this may be geographically variable, e.g. this is not consistent in Argentina^11^. The strains are morphologically identical, but can be distinguished using DNA barcodes^12^ which show two distinct clusters^13^ that may have diverged 2 myr ago and now have a mean sequence divergence of 2.09%^14^. The maize strain shows additional population structure when the ratios of four slightly different barcode haplotypes are examined at the population level: the population based in Florida and the Caribbean (Florida haplotype profile) differs from that found from Texas through Central America to Argentina (Texas haplotype profile)^15^. There is limited genetic exchange between them so that each may have acquired different biological characteristics, e.g. resistance to pesticides or GM maize. The rice and corn strains each have a separate Barcode Index Number (BIN^16^), *S*. *frugiperda* (rice strain) being BOLD: ACE4783, and *S*. *frugiperda* (maize strain) being BOLD: AAA4532. The two species or strains are sympatric, continuously breeding from southern USA to northern Argentina, and both occur as temporary breeding immigrant populations further north and further south during summer and autumn, but are unable to tolerate freezing temperatures. Whether these two clusters represent two interbreeding races, two separating species, two separated populations that are merging, or two separate species is not yet entirely clear, but the most recent studies^14, 17, 18^ incline towards the last view, with reproductive isolation between the two species in at least part of their range. However, it may be premature to assume that this condition holds throughout the range of both species, although, given their vagility, this may well be the case. If they are accepted as species, as yet it is not clear which species would be the true *S*. *frugiperda*, or to which species the five accepted synonyms^6^ of *S*. *frugiperda* apply. Types are available or have been designated for all names, but specimens date back to the nineteenth century, apart from the 1996 neotype of *S*. *frugiperda* ^6^, so barcoding this old material will be challenging. Here, for clarity we choose to refer to the two barcode clusters as species: *S*. *frugiperda* sp. 1 (ACE4782) (appearing in BOLD and literature as *S*. *frugiperda* sp. 1 haplotype 1, rice strain, R strain, DHJ01) and *S*. *frugiperda* sp. 2 (AAA4532) (appearing in BOLD and literature as *S*. *frugiperda* sp. 2., haplotype 2, maize strain, corn strain, C strain, DHJ02).

*Spodoptera frugiperda* was recently reported from Africa for the first time^3^, on the mainland of West Africa (Nigeria, Togo, Benin) and from the island of São Tomé (São Tomé and Príncipe). Four specimens barcoded from Nigeria matched *S*. *frugiperda* sp. 2 (AAA4532) and two from São Tomé matched *S*. *frugiperda* sp. 1 (ACE4782). *Spodoptera frugiperda* sp. 1 (ACE4782) has not hitherto been recorded from mainland Africa.

Since 2012 the CABI-led initiative, Plantwise (www.plantwise.org) has been supporting the Plant Protection and Regulatory Services Directorate of Ghana (PPRSD) to strengthen the plant health system in Ghana, by promoting linkages with other stakeholders in the plant health system, training plant doctors, establishing plant clinics, developing extension information and carrying out mass extension for plant health issues^19^. In early 2016 the PPRSD first became aware of an apparently new type of armyworm damage on maize in parts of Eastern and Volta regions of the country. Almost all the reports received were from extension staff trained as plant doctors under the Plantwise programme. Apart from reporting directly to the national office, plant doctors also used the WhatsApp TM and the Telegram TM messaging apps to report the pest and solicit input of other plant doctors on management practices. Photographic images of the pest sent to CABI UK by a Plantwise plant doctor were not sufficient alone to substantiate an identification of this New World species as a new pest for Ghana and the plant doctor was asked to provide further images and preserved adult specimens if possible, and the PPRSD became aware of this potential new pest at this time. The plant doctors were unable to supply adult specimens of the pest, but similar pest damage was later reported in other parts of the country: Brong Ahafo, Greater Accra, Ashanti, Central, Northern, Upper East and Upper West regions, but not as yet (December 2016) from the Western region. Thus, Plantwise played a key supporting role in detection, raising the alarm and identification of this new pest. The PPRSD together with CABI Plantwise staff set out to establish the causative agent. Here we report the results of that investigation.

## Results

### Field collections

**Figure 1:**
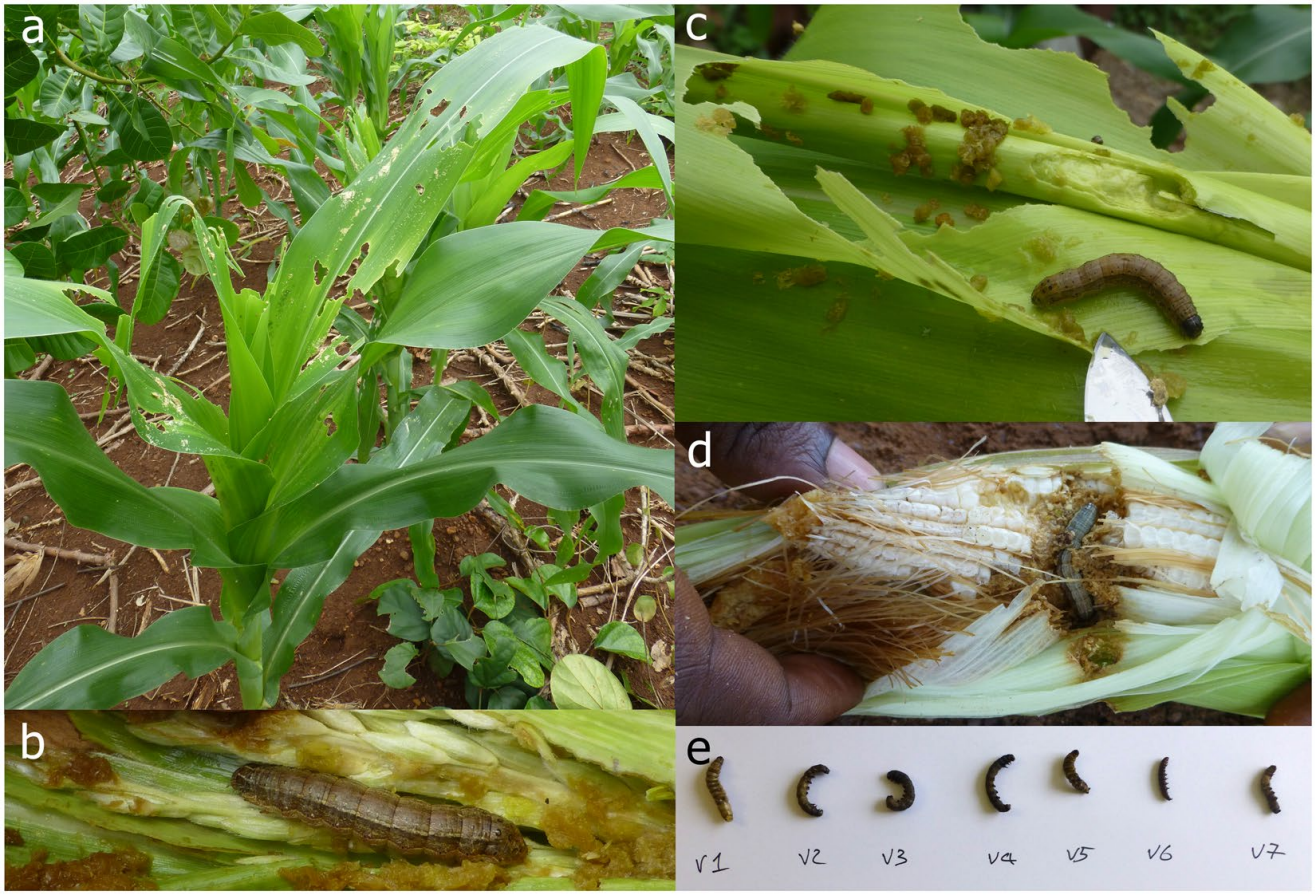
Field observations. Images of (**a**) damage, (**b**–**d**) live larvae and (**e**) preserved larvae from field work. This type of material alone did not justify the identification of *Spodoptera frugiperda* as a new pest species for Ghana, and so molecular methods were needed. (All images: Jayne Crozier).

Damage to maize was investigated at Keta and Anfoeta (Volta), Techiman, Ayeasu, Jema, Nante, Kintampo, Chiranda (Brong Ahafo) and Tamale (Northern). Larvae were associated with the observed symptoms, photographed and collected at all of these locations (Fig. 1). Larvae were also collected from maize samples brought into Plant Clinics by farmers in Sognayili, Savelugu and Kepene located close to Tamale in the Northern region. There were larvae of two different phenotypes: relatively thick green-brown caterpillars which were associated with the new damage symptoms, and smaller dark brown larvae which may have been younger individuals of the same species or something different.

### Molecular identification and analysis

The barcodes obtained from our samples were compared with public barcodes in BOLD and GenBank, and shown to comprise a mixture of both species of *Spodoptera frugiperda* and *Busseola fusca* (Fuller) (Noctuidae) (Table 1).

**Table 1:** GenBank accession numbers and identifications.

Isolate	Source Region	GenBank accession number for COI barcode	Identification
CABI-AWB01	Brong Ahafo	KY472239	*Busseola fusca*
CABI-AWB02	Brong Ahafo	KY472240	*S*. *frugiperda* sp. 1 (ACE4782)
CABI-AWB03	Brong Ahafo	KY472241	*S*. *frugiperda* sp. 1 (ACE4782)
CABI-AWB04	Brong Ahafo	KY472242	*S*. *frugiperda* sp. 1 (ACE4782)
CABI-AWB05	Brong Ahafo	KY472243	*Busseola fusca*
CABI-AWB09	Brong Ahafo	KY472244	*S*. *frugiperda* sp. 1 (ACE4782)
CABI-AWB10	Brong Ahafo	KY472245	*S*. *frugiperda* sp. 1 (ACE4782)
CABI-AWB11	Brong Ahafo	KY472246	*Busseola fusca*
CABI-AWB13	Brong Ahafo	KY472247	*Busseola fusca*
CABI-AWN01	Northern Region	KY472248	*S*. *frugiperda* sp. 2 (AAA4532)
CABI-AWN03	Northern Region	KY472249	*S*. *frugiperda* sp. 1 (ACE4782)
CABI-AWN05	Northern Region	KY472250	*S*. *frugiperda* sp. 1 (ACE4782)
CABI-AWV01	Volta Region	KY472251	*S*. *frugiperda* sp. 2 (AAA4532)
CABI-AWV03	Volta Region	KY472252	*S*. *frugiperda* sp. 2 (AAA4532)
CABI-AWV04	Volta Region	KY472253	*S*. *frugiperda* sp. 1
CABI-AWV05	Volta Region	KY472254	*S*. *frugiperda* sp. 2 (AAA4532)
CABI-AWV06	Volta Region	KY472255	*S*. *frugiperda* sp. 2

Details are given of the CO1 barcodes obtained from samples of Lepidoptera larvae collected from maize in Ghana, 26 September–7 October 2016.

**Figure 2:**
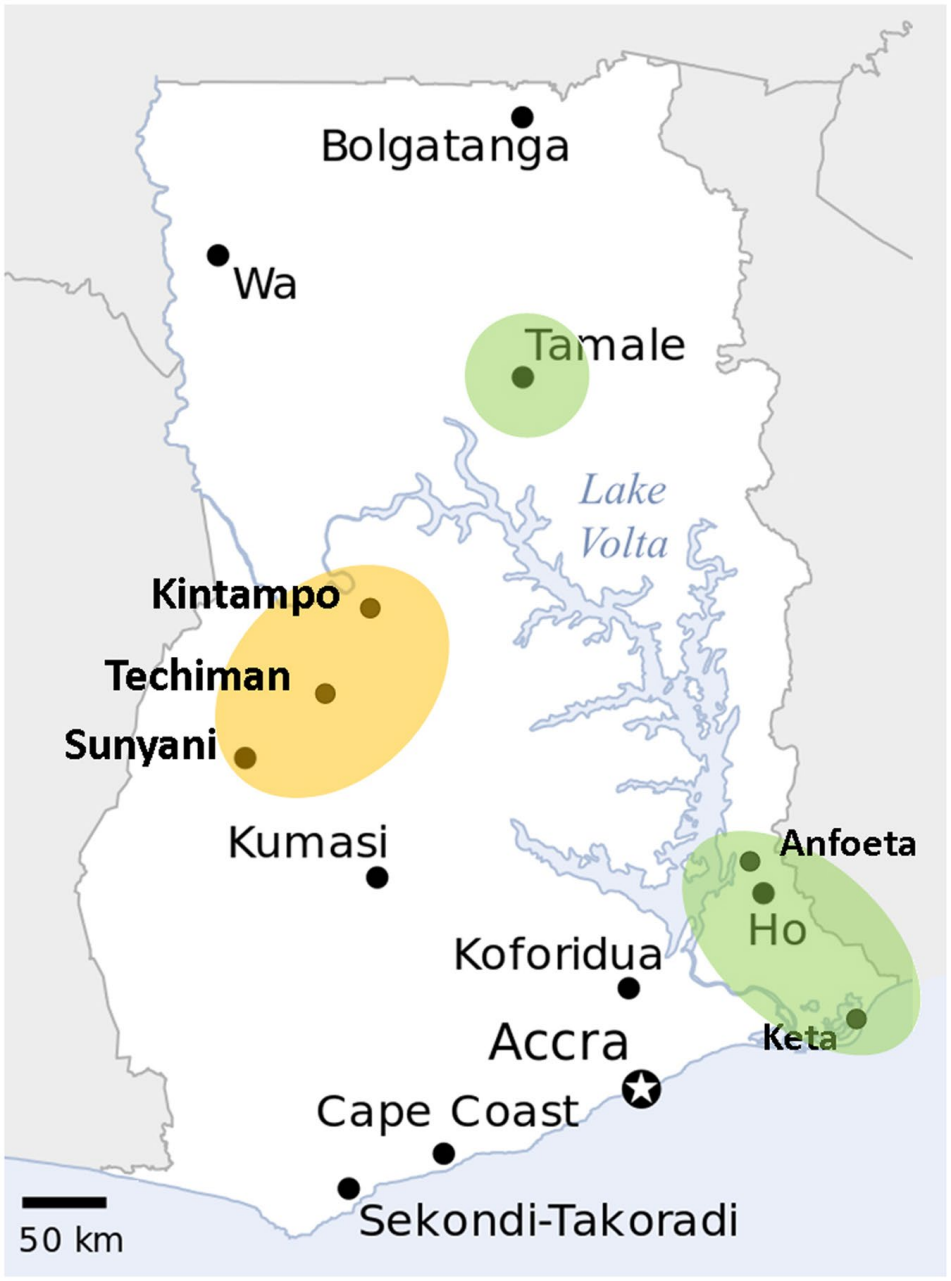
Survey results. Map of Ghana showing three survey locations (highlighted), major towns and towns closest to collection sites. Both species of *Spodoptera frugiperda* were found in samples from Keta and Anfoeta (Volta), and Tamale (Northern), shaded green, but only *S*. *frugiperda* sp. 1 (ACE4782) from collections around Techiman and five nearby communities (Brong Ahafo), shaded brown. Small larvae of *Busseola fusca* were also collected in the Brong Ahafo samples. Based on an OCHA/ReliefWeb created by the UN Office for the Coordination of Humanitarian Affairs (OCHA), downloaded from Wikipedia (https://commons.wikimedia.org/wiki/File:Ghana_-_Location_Map_(2013)_-_GHA_-_UNOCHA.svg) under a CC BY 3.0 license, and edited using Microsoft Publisher TM and Adobe Photoshop Elements TM.

As noted above, larvae of two different phenotypes were collected: relatively thick green-brown caterpillars which were associated with the new damage symptoms, and smaller dark brown larvae which may have been younger individuals of the same species or something different. The head and body of larvae of *S*. *frugiperda* are known to show individual variation in colour^4, 6^, so the two types found in field collections could not be reliably identified by eye. Barcoding showed that the former conformed to one or other species of *S*. *frugiperda* and the latter were the maize stem borer, *Busseola fusca*. The distribution of confirmed records is shown in Fig. 2.

### Phylogenetic analysis

**Figure 3:**
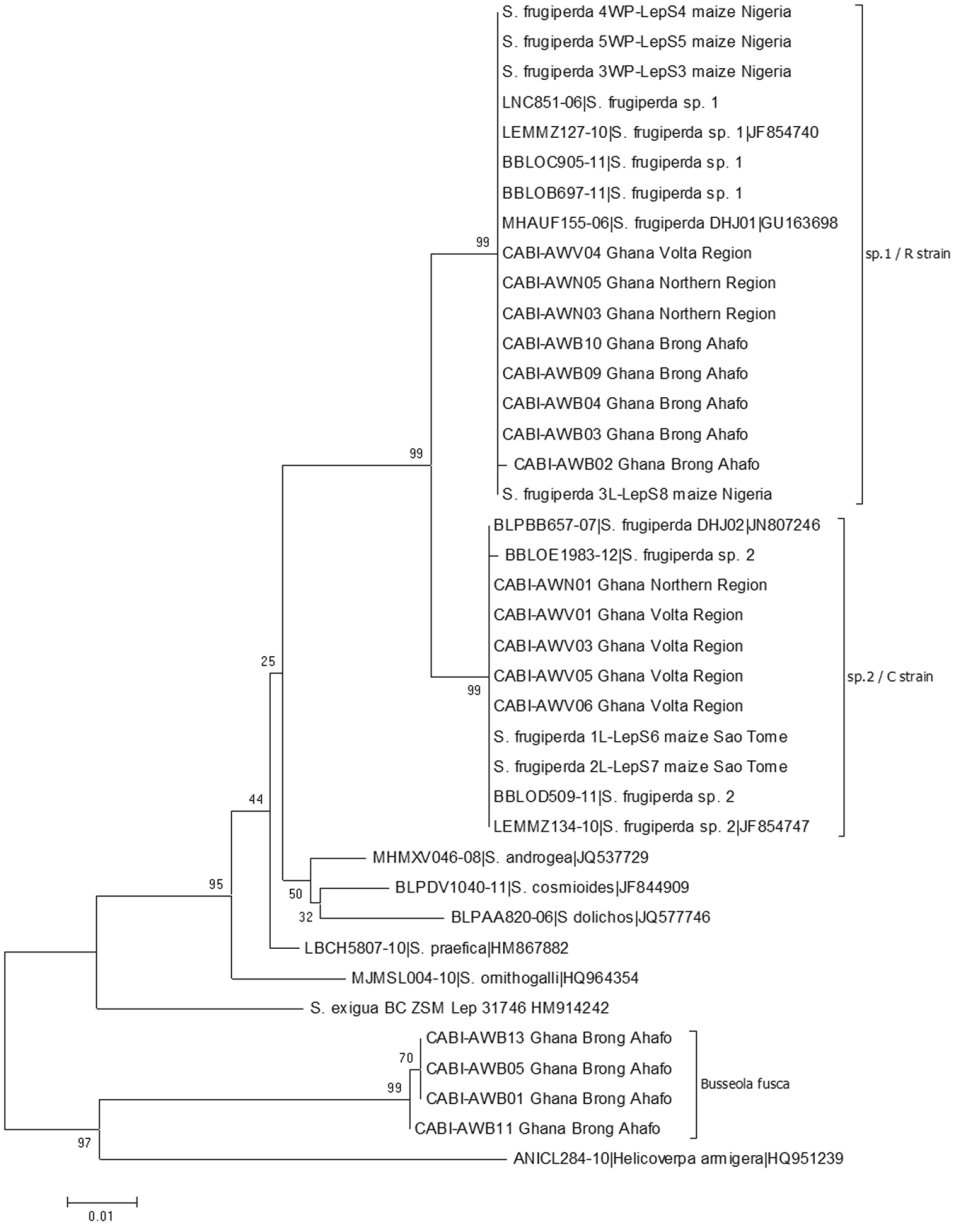
Phylogeny of African *Spodoptera frugiperda*. This tree includes all available barcodes of African samples and selected American samples to show the relationship between the two species of *S*. *frugiperda* and other *Spodoptera* spp. based on Supplementary Figure 2. Our samples are coded ‘CABI-’.

In support of these identifications we also made two phylogenetic analyses. Firstly we constructed a tree combining all publicly available authenticated barcodes of the *S*. *frugiperda* complex present in BOLD (http://www.boldsystems.org/), the recent African barcodes deposited in GenBank by Goergen *et al*.^3^ plus the new barcodes obtained in the present study, and our samples of *Busseola fusca* as outgroup (Supplementary Figure 1). This confirmed that *S*. *frugiperda* divides into two clear clusters, with very little barcode variation in each, and that the barcodes of our samples were identical to those reported from the Americas and elsewhere in Africa. Eight of the 13 (61.5%) *S*. *frugiperda* samples that we obtained conformed to ‘sp. 1’ whilst the remaining five sequences (38.5%) matched the ‘sp. 2’ barcode. We observed no evidence of any hybrid form and have found no record in the literature of any such hybrid. We then took selected samples of the *S*. *frugiperda* complex including ours, those recently reported from Africa by Goergen *et al*.^3^, and representative samples of the two species from across its American distribution (Supplementary Figure 2). This showed a well-structured tree inasmuch as the species were clearly defined, but the phylogenetic structure was only weakly supported. Nevertheless, the difference between the two species of S. *frugiperda* was strongly supported and the gap between them smaller, but comparable to that between other species pairs that are phenotypically different. We include Fig. 3 to illustrate the two *S*. *frugiperda* species and their nearest neighbours on this tree.

## Discussion

The analysis of our collections from three regions in Ghana has shown that both species of *S*. *frugiperda* are widespread attacking maize, although we did not find *S*. *frugiperda* sp. 2 (AAA4532) in the five *S*. *frugiperda* samples sequenced from Brong Ahafo. Based on our results, *S*. *frugiperda* has now been reported to the International Plant Protection Convention as present in some areas of Ghana^20^. The chronology of initial reports in Ghana gives a strong indication that the pest spread from the east to other parts of the country, and so probably entered Ghana from across the border with Togo, where it was reported in 2016^3^. The initial report by Goergen *et al*.^3^ found *S*. *frugiperda* sp. 1 (ACE4782) in São Tomé (two specimens barcoded) and *S*. *frugiperda* sp. 2 (AAA4532) in Nigeria (four specimens barcoded), while the records from Togo and Benin were not based on barcoding. It seems the more likely scenario that both species have been present in mainland Africa all along, rather than *S*. *frugiperda* sp. 1 (ACE4782) has spread from an initial establishment on São Tomé to the mainland. Barcoding other dated material from Nigeria, Togo and Benin should clarify this. Since the record from São Tomé was based on only two barcodes, the possibility that both species are present there should also be investigated.

We considered the possibilities underlying the current situation: is *S*. *frugiperda* spreading very rapidly in mainland Africa or has it been present and overlooked for some years? Given what we know about its vagility in the Americas, and that the conspicuous new damage to maize cobs in the field was easily detected and recognised as new by extension staff, we think it more likely that *S*. *frugiperda* is spreading very rapidly, and it can be expected to spread to the limits of suitable African habitat within a few years. The African maize-growing countries may plan for this by preparing alerts for extension services and researchers, and assessing advice on the best management options for farmers. In Ghana, a poster and a flyer to facilitate identification^21, 22^, and a pest management decision guide^23^ to inform extension staff have been prepared. These information aids are being disseminated in hard copy and through the Plantwise knowledge bank (http://www.plantwise.org/KnowledgeBank/), and awareness-raising activities to inform farmers are ongoing. Although *S*. *frugiperda* is known to be polyphagous in the Americas, affecting many crops, especially Poaceae^4, 6^, little evidence for this has come to light so far in Ghana, apart from some reports from cowpea and groundnuts. It will be important to assess the threat that *S*. *frugiperda* presents to other crops in Africa, and the implications that of the use of other crops may have for the population dynamics of the pest. In the first place, extension workers and farmers will need to recognise and report *S*. *frugiperda* damage on other crops, so the larval identification guides^21, 22^ will be important. Further research will be needed on these aspects.

At the moment, the two species seem to be spreading more or less together. Although we do not know the exact American source of the introduction(s) into mainland Africa, or what the genetics of the two *S*. *frugiperda* species are in that American source area, e.g. whether the population of *S*. *frugiperda* sp. 2 (AAA4532) introduced into Africa are from the Florida or Texas haplotype profile^15^, we should anticipate that the introduced population has gone through a genetic bottleneck during the introduction and establishment phase. It is possible this may have led to changes in the dynamics of hybridization, so that no assumptions should be made about the relationship of the two *S*. *frugiperda* species, and their isolation, or otherwise, in Africa. All behaviours observed in the Americas can be anticipated until more is known about the two species as the pest spreads in Africa. For example, both introduced *S*. *frugiperda* species in Africa have been found attacking maize, but we do not yet have data on the wider host range in Africa, and whether this segregates by *S*. *frugiperda* species.

As Goergen *et al*.^3^ commented, the original introduction or introductions must have involved at least one female of each *S*. *frugiperda* species. Because intercontinental introductions like this are rare events, and this is the first recorded occurrence of *S*. *frugiperda* in Africa, we think it more likely that the two *S*. *frugiperda* species were introduced together than that there were separate introduction events for each *S*. *frugiperda* species at more or less the same time. Introduction may have been as eggs, caterpillars, pupae or adults, or any combination of these. We consider possible pathways of introduction in the context of the framework put forward by Hulme *et al*.^24^. Of the six possible types of pathway recognised, only three might have been applicable in this case: unaided dispersal, contaminant of a commodity and stowaway on a vector.

Adults fly actively and, as noted, regularly move over long distances with air currents before oviposition; however, the prevailing trade winds are from Africa to the Americas making unaided dispersal by adult flight a very unlikely pathway of entry in this case. Furthermore, if it were a possibility, it seems unlikely that it would not have happened before, perhaps long ago.

Transfer as a contaminant of a commodity, e.g. fresh produce is a possibility. In an analysis of quarantine interceptions into the USA, 1984–2000, McCollough *et al*.^25^ found that insects in cargo were most frequently intercepted on cut flowers, plant parts and fruit (in rank), whereas insects in baggage were most frequent on fruit, plant parts, seed and cut flowers (in rank). Given their feeding habits, larvae of *S*. *frugiperda* are most likely to be transferred from the Americas within plant parts, e.g. a maize cob with the sheath in place. Pupation is normally in the soil, but could be amongst plant material if confined, e.g. a bag of fresh, infested produce. Both of these scenarios are possible with modern air transport and travel. Analysis of the interceptions on plant produce coming into the European Union and Switzerland, 2012–2016^26^, revealed an average of 7.2 interceptions of *S*. *frugiperda* per year, of which 17 were on capsicum peppers, 11 on other *Solanum* spp. and 8 on parts of other plants. Suriname was the commonest source country (26 interceptions), but *S*. *frugiperda* was also intercepted from Dominican Republic, Ecuador, Guatemala, Mexico, and Peru, but not from the USA. Compared to Europe, the cargo importation of fresh produce known to harbour early stages of *S*. *frugiperda* from the Americas into Africa is extremely limited, estimated at less than 10 tonnes per year^27^. No consolidated data is available on how much and what type of produce is carried in passenger baggage, nor on interceptions of insect pests at African ports. The combination of phytosanitary precautions and minimal trade in fresh produce between Africa and the Americas indicates that the chances of transferring viable numbers of both *S*. *frugiperda* species as contaminants are extremely small.

On balance we consider the chances of a successful transfer as a stowaway on a direct flight seem significantly more likely. Eggs are laid in tightly packed groups of from a few to hundreds of eggs, and covered with scales from the end of the female’s abdomen^28^; these egg masses are normally laid on the food plants, but can be laid indiscriminately, including on inorganic substrates, especially when populations are high^28, 29^. The newly hatched caterpillars disperse by walking and on the wind, ballooning on silk threads, before starting to feed on host plants. Egg masses can be laid in, or on, parts of aircraft, including wheel bays. In one 1950 study^30^, more than 9,000 aircraft coming from South America and the Caribbean were examined at Miami airport; Lepidoptera eggs were found on 98 of these (0.86%), and the predominant species was *S*. *frugiperda*. The number of egg masses on each plane varied from one to about 1000. Survival of insects on intercontinental flights may be high^31^ and would be excellent on cargo containers transferred within a pressurised hold. For eggs to be the means of transfer, it would be necessary after arrival for the aircraft — or whatever part of its equipment had eggs on it — to be placed close to, and upwind from, suitable food plants, thereby enabling newly hatching caterpillars to be carried to them on the wind.

Alternatively, pre-oviposition female moths could settle in parts of an aircraft such as the cargo holds or wheel bays, and this also seems a possible mechanism for transfer. Transfer of adults and eggs is most likely to occur on a direct flight; for example, currently (December 2016) there are direct commercial flights between Atlanta (Georgia, USA) and Lagos (Nigeria), and between São Paulo (Brazil) and Lomé (Togo). Analysis of a larger sample from the introduced population in Africa, may throw light on the origin, e.g. evidence of the Florida haplotype profile would suggest an eastern North America origin rather than a South American origin. A more definitive answer would require comparative (mitochondrial) genomics between examples of both species in order to see if the differences seen in the COI barcode region are reflected in differences in other functional genes.

Onward spread within Africa is already happening and there are widespread reports in the press and on-line from more than ten countries in central, eastern and southern Africa, although only those from South Africa, Swaziland and Zambia had been formally confirmed with the International Plant Protection Convention by the end of February 2017^32–34^. We have no evidence regarding the methods of spread within Africa, but it seems likely that unaided dispersal by flight, contaminant of nationally and internationally traded commodities and stowaway on airplane and vehicle vectors all play a role. Indeed recent reported outbreaks in southern Africa raise the question as to how long *S*. *frugiperda* has been present in this region. Given that the climate is more seasonal and there are marked dry seasons, *S*. *frugiperda* is unlikely to be breeding continuously, unlike much of West Africa, and so may have taken several years to build up to outbreaks. Hence it is not impossible that the original reports in West Africa do not represent the first introductions into the continent.

Traditionally, identification of Lepidoptera is based on characters of the adults, and not the damaging caterpillar stage^35^. Detecting and identifying a new Lepidoptera pest has involved collecting caterpillars, rearing them through to adults, pinning and spreading adults to facilitate identification, and often dissection of the male and/or female genitalia to confirm an identification. This work is best carried out by experienced entomologists, preferable those familiar with working with Lepidoptera. In our approach, we did not have this luxury, and the field team comprised a plant pathologist from the Plantwise programme (J.C.) and a national plant protection officer (P.B.); the team took photographs of the caterpillars (Fig. 1b–d) and the damage (Fig. 1a) and preserved samples of the caterpillars in ethanol (Fig. 1e). These caterpillar samples were not suitable material from which to make an authoritative identification, but were collected because we knew they could be barcoded^13^ and that authenticated barcodes were publicly available for most armyworm pests (BOLD; http://www.boldsystems.org/ ^36^) and against which we could compare our new barcodes. The approach worked as planned. An added benefit, which we had not explicitly anticipated is that barcoding could also be used to identify young caterpillars (in this case *Busseola fusca*), which are not as easy to diagnose as those in the final instar.

The current study has implications for future pest diagnosis and identification, particularly for invading or new pests in developing countries. Currently, extension and research staff in most developing countries rely on limited in-country capacity for identification of pest problems (e.g. as documented by Mugambi *et al*.^37^ in Kenya), occasionally with external support through programmes such as Plantwise or international agricultural research centres^3^. The molecular methods for barcoding specimens are becoming more readily available and affordable in most countries. It would be feasible to determine the species/haplotype present using a simple method such as COI RFLPs which, subject to use of an appropriate restriction endonuclease, could allow discrimination of the species/haplotypes on the basis of fragment size. However, in common with all such fragment-based methods, one would gain no information regarding the sample – other than the RFLP fragment sizes (and the sequence at the beginning and end of each fragment – which would match the cutting site of the restriction endonuclease). This is one of the main reasons why RFLP techniques fell out of favour at the end of the last century^38^. We wished to obtain definitive, unequivocal evidence of the species/haplotype in each case for which direct sequencing was the only option. Indeed, the fact that we only discovered we had samples of *Busseola fusca* due to our sequence analysis, shows the necessity of obtaining sequence data and not relying on RFLP band sizes or profiles. In future, the approach employed in the present study can be used to identify problem pests in countries, providing that there is a comprehensive public library of barcodes of the world’s pests available^39^. This is not yet the case, but we are moving rapidly towards this ideal, and for the most important pests, especially Lepidoptera, and particularly those Lepidoptera that also occur in developed countries, this approach will already work. It is noteworthy that almost the entire Lepidoptera fauna of north-western Costa Rica, including *S*. *frugiperda*, has been barcoded^40^ so that any species that occurs there can be provisionally identified elsewhere; this is an example where basic ecological research is generating direct benefits for economic pest detection. There will always be a critical need for morpho-taxonomic expertise for newly discovered/recognised taxa but molecular methods can build upon the resources available for known characterised taxa. DNA Barcoding, therefore, will facilitate the in-country identification of pests in future, but will also effectively focus taxonomic support to where it is needed: i.e. species with no reference barcode and which, as a result, require targeted specialist help to enable authoritative identification; and situations where pest species are (or have been) confused with morphologically similar taxa, or in cases where the problem may comprise more than one species (as here).

## Methods

### Survey methods

The survey to collect samples was not systematically carried out as the prime objective was to establish the causative agent of recent reports of what appeared to be an unknown armyworm species (at that time Goergen *et al*.^3^ were yet to publish their findings). Whilst on a mission to complete a review of monitoring of plant clinic performance in the participating regions of Ghana, 26 September–7 October 2016, the Plantwise team were alerted to the severity of the pest outbreak and collected samples at the roadside where symptoms on maize crops were evident. Symptoms included severe feeding damage on maize leaves with numerous holes and ragged edges, on closer inspection larvae and frass were found associated with feeding in the funnels and inside the cobs. Where symptoms were observed, samples were collected from maize close to the roadside around Techiman, Jema, Nante, Kintampo, Chiranda, Ayeasu and Sunyani in Brong Ahafo Region, Keta and Anfoeta in the Volta Region, Tamale, Savelugu, Sognayili and Kpene in the Northern Region. Further samples were collected from maize plants brought to Plant Clinics by farmers around Tamale and Sunyani. The samples from each location were placed in a single sterile microcentrifuge tube containing 70% ethanol for transport to the laboratory freezer in the UK.

### Molecular identification and analysis

DNA samples were stored at −20 °C for at least 24 hours before being further processed. A fragment of the abdomen of each specimen was air-dried for 5 minutes, then rinsed with 50 µl sterile molecular grade H_2_O (ThermoFisher Scientific, UK) to rehydrate the sample and to dilute residual ethanol. Excess water was removed, and DNA templates for PCR amplification were obtained by adding 20 µl of microLYSIS®-PLUS (MLP; Microzone Ltd., UK) to the dried material. The suspension was macerated with a sterile micropestle (VWR International Ltd., UK) to facilitate the disruption of the exoskeleton and tissues of the samples. DNA was then liberated into the MLP by placing the sample tubes in a thermal cycler and subjecting to the heat profile recommended by the manufacturer, for difficult samples.

PCR reactions were carried out using a Hybaid PCR Express thermal cycler in heated-lid mode. Amplifications were carried out in 0.5 ml microcentrifuge tubes in 20 µl reactions containing: 1 µl MLP DNA extract; Primers LCO1490 and HCO2198 (5′-GGTCAACAAATCATAAAGATATTGG-3′ and 5′-TAAACTTCAGGGTGACCAAAAAATCA-3′, respectively^41^) each at 150 nM; and 10 μl of MegaMix-Royal (Microzone Ltd, UK) mastermix solution, containing optimised mixture of *Taq* polymerase in 2 × Enhancing Buffer (6 mM MgCl_2_), with 400 μM dNTPs and blue MiZN loading dye. Reactions were made up to a final volume of 20 μl sterile molecular grade H_2_O. PCR reactions were preincubated for 5 min at 95 °C followed by 39 cycles of: 30 s at 94 °C; 30 s at 51 °C; 75 s at 72 °C. Samples were finally incubated for 10 min at 72 °C followed by chilling at 10 °C. In accordance with our standard practice, a ‘no DNA’ negative control (components as above but containing 1 µl sterile H_2_O instead of DNA) was included with each set of reactions.

Where necessary, a second round of amplification (i.e. ‘reamplification’) was undertaken as follows: 1 µl of each of the above PCR products was used as template. The reaction was carried out under the same conditions, with the exception of the number of cycles, which was reduced to 30. In such cases, a fresh ‘no DNA’ negative control was prepared as described previously but an additional negative control was prepared using 1 µl of the first round ‘no DNA’ negative control reaction mix for that reaction set. Aliquots (4 µl) of each PCR product were used for agarose gel electrophoresis with 1.5% (w/v) Hi-Pure Low EEO agarose (BioGene Ltd, UK) in 0.5x TBE (Severn Biotech Ltd, UK) running buffer, containing 5 µl SafeView nucleic acid stain (NBS Biologicals Ltd., UK) for 100 ml of 0.5X TBE, and with 4 µl 100 bp size marker (ThermoFisher Scientific, UK). PCR products of the expected size (ca. 650 bp; see Supplementary Figures 3 and 4 [It may be noted, also, from Supplementary Figures 3 and 4 that there was no visible amplification of first and/or second round ‘no DNA’ negative controls, thereby showing that any positive reactions obtained were genuine and not artefactual or contaminant in nature]) were purified using microCLEAN purification solution (Microzone Ltd., UK) in accordance with the manufacturer’s instructions. Purified products were resuspended in 15 µl sterile molecular grade H_2_O.

Sequencing of PCR products was undertaken using a thermal cycler (MWG Primus, Germany) in heated-lid mode with BigDye® Terminator v3.1 cycle sequencing kit (ThermoFisher Scientific, UK). Sequencing reactions contained the following, in 0.5 ml microcentrifuge tubes: 2.68 µl of template DNA prepared as above; Primer HCO2198 at 320 nM; 5x BigDye® Terminator Sequencing Buffer; BigDye® Terminator. The sequencing reactions were preincubated for 1 min at 96 °C followed by 25 cycles of: 20 s at 96 °C; 10 s at 50 °C; 4 min at 60 °C. Samples were finally chilled at 10 °C. Excess unincorporated dye-terminators were removed using DyeEx® 2.0 spin columns (Qiagen, UK) according to the manufacturer’s recommendations, with the eluted purified sequencing reaction products being resuspended in 16 µl of Hi-Di ^TM^ formamide (ThermoFisher Scientific, UK) prior to automated capillary electrophoresis and sequence reading on an ABI 3130 Genetic Analyser (ThermoFisher Scientific, UK). Sequences obtained after a second round PCR ‘reamplification’ were of as good quality as those obtained from a single round PCR. Samples were only considered to be positive for FAW (or, indeed, *B*. *fusca*) if they gave a good quality sequence – i.e. appearance of a band was not sufficient. This enabled us to ensure that our results were not artefactual, contaminant or chimaeric. PCR success rates were at 60–71% (i.e. nine sequences obtained from 13 samples from Brong Ahafo [69% successful]; 3/5 from Northern Region [60%]; 5/7 from Volta Region [71%]). Sequences were aligned using the multiple sequence alignment plug-in CLUSTALW in MEGA6^42^. Sequences obtained in the present study were compared with authenticated sequences obtained from the Barcoding of Life Data system (BOLD; http://www.boldsystems.org/ ^16^) and additional sequences from the GenBank® data base (http://www.ncbi.nlm.nih.gov/genbank/^43^). Alignment used the default parameters of CLUSTALW^44^ and MUSCLE^45, 46^ and these were then optimized manually in the MEGA6 program^42^.

### Phylogenetic analysis

Inference of relationships was by the maximum likelihood (ML) method in MEGA6^42^. Branch support was estimated by bootstrap analysis (1000 replicates). The evolutionary history was inferred by using the Maximum Likelihood method based on the Tamura-Nei model^47^. The tree with the highest log likelihood (−1795.3808) is shown as Fig. 3. The percentage of trees in which the associated taxa clustered together is shown next to the branches. Initial tree(s) for the heuristic search were obtained automatically by applying Neighbor-Join and BioNJ algorithms to a matrix of pairwise distances estimated using the Maximum Composite Likelihood (MCL) approach, and then selecting the topology with superior log likelihood value. The tree is drawn to scale, with branch lengths measured in the number of substitutions per site. The analysis involved 39 nucleotide sequences. Codon positions included were 1^st^ + 2^nd^ + 3^rd^ + Noncoding. All positions with less than 95% site coverage were eliminated. That is, fewer than 5% alignment gaps, missing data, and ambiguous bases were allowed at any position. There were a total of 624 positions in the final dataset. Further phylogenetic and evolutionary analyses were conducted in MEGA6^42^. Additional trees are shown in Supplementary Figures 1 and 2.

Noctuoid classification has been in a state of change in recent years, as molecular evidence has been used to develop the phylogeny. *Spodoptera* is the only genus in the tribe Prodeniini, but the placement of this tribe amongst the subfamilies of Noctuidae is not clear. One of the most recent studies^48^ showed that it does not belong in Noctuinae as previously thought, and its closest relatives seem to be Heliothinae, and so in addition to other *Spodoptera* spp. we included *Helicoverpa armigera* (Hübner) and our samples of *B*. *fusca* as outgroups.

### Data Availability

The datasets generated during and/or analysed during the current study are available from the corresponding author on reasonable request. All DNA barcode sequences obtained have been deposited at NCBI GenBank with the accession numbers KY472239-KY472255.

## Electronic supplementary material

Supplementary material
