# Supplementary material for: Molecular methods to detect Spodoptera frugiperda in Ghana, and implications for monitoring the spread of invasive species in developing countries
Source: Sci Rep. 2017 Jun 22;7:4103. doi: 10.1038/s41598-017-04238-y (PMC5481405; doi:10.1038/s41598-017-04238-y)
Supplement: Supplementary file 1 — Supplementary material [file 41598_2017_4238_MOESM1_ESM.doc]

**Molecular methods to detect *Spodoptera frugiperda* in Ghana, and implications for monitoring the spread of invasive species in developing countries**

Matthew J.W. Cock^1^, Patrick K. Beseh2^2^, Alan G. Buddie^1^ (corresponding author), Giovanni Cafá^1^ & Jayne Crozier^1^

^1^CABI, Bakeham Lane, Egham, TW20 9TY, UK, ^2^MOFA-PPRSD, P.O. Box M37, Accra, Ghana. Correspondence and requests for materials should be addressed to A.G.B. (email: a.buddie@cabi.org)

**Supplementary Material**

**Supplementary Figure 1. Molecular Phylogenetic analysis by Maximum Likelihood method.** The evolutionary history was inferred by using the Maximum Likelihood method based on the General Time Reversible model^1^. The tree with the highest log likelihood (-1189.2763) is shown. The percentage of trees in which the associated taxa clustered together is shown next to the branches. Initial tree(s) for the heuristic search were obtained by applying the Neighbor-Joining method to a matrix of pairwise distances estimated using the Maximum Composite Likelihood (MCL) approach. The tree is drawn to scale, with branch lengths measured in the number of substitutions per site. The analysis involved 114 nucleotide sequences. All positions with less than 95% site coverage were eliminated. That is, fewer than 5% alignment gaps, missing data, and ambiguous bases were allowed at any position. There were a total of 628 positions in the final dataset. All *S. frugiperda* barcodes available in BOLD and >620bp were included. Sample labels are as given in the BOLD database. Evolutionary analyses were conducted in MEGA6^2^.

**Supplementary Figure 2. Molecular phylogenetic analysis by maximum likelihood method.** The evolutionary history was inferred by using the Maximum Likelihood method based on the General Time Reversible model^1^. The tree with the highest log likelihood (-2895.8236) is shown. The percentage of trees in which the associated taxa clustered together is shown next to the branches. Initial tree(s) for the heuristic search were obtained automatically by applying Neighbor-Join and BioNJ algorithms to a matrix of pairwise distances estimated using the Maximum Composite Likelihood (MCL) approach, and then selecting the topology with superior log likelihood value. The tree is drawn to scale, with branch lengths measured in the number of substitutions per site. The analysis involved 62 nucleotide sequences. Codon positions included were 1st+2nd+3rd+Noncoding. All positions with less than 95% site coverage were eliminated. That is, fewer than 5% alignment gaps, missing data, and ambiguous bases were allowed at any position. There were a total of 628 positions in the final dataset. Sample labels are as given in the BOLD database. Evolutionary analyses were conducted in MEGA6^2^.

1. Nei, M. & Kumar, S. *Molecular Evolution and Phylogenetics*. Oxford University Press, New York (2000)

2. Tamura, K., Stecher, G., Peterson, D., Filipski, A. & Kumar S. MEGA6: Molecular Evolutionary Genetics Analysis version 6.0. *Molec. Biol. Evol.* **30,** 2725-2729 (2013)


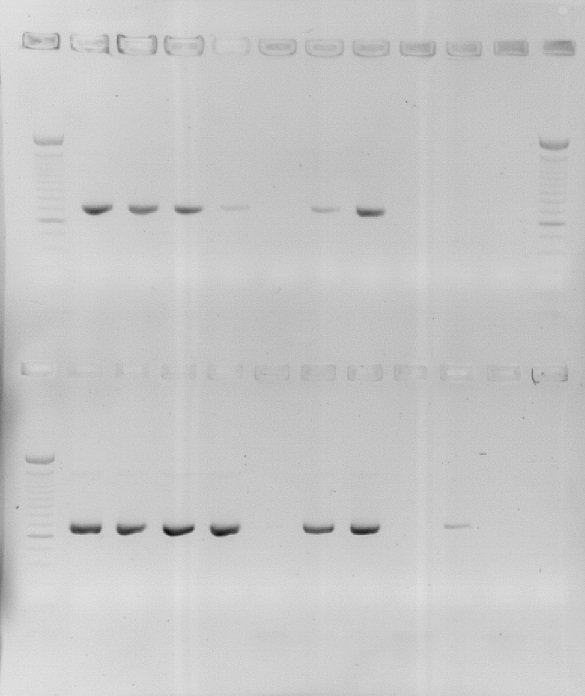


**Supplementary Figure 3**. Gel Electrophoresis of the products of amplification of mitochondrial cytochrome c oxidase subunit I from DNA extracts of armyworms. In the top lane, samples were loaded in the following order: 100bp size marker, CABI-AWB01, CABI-AWB02, CABI-AWB03, CABI-AWN01, CABI-AWN02, CABI-AWN03, CABI-AWV01, CABI-AWV02, CABI-AWV03, No template control, 100bp size marker. In the bottom lane, samples were loaded in the following order: 100bp size marker, CABI-AWB01-Re, CABI-AWB02-Re, CABI-AWB03-Re, CABI-AWN01-Re, CABI-AWN02-Re, CABI-AWN03-Re, CABI-AWV01-Re, CABI-AWV02-Re, CABI-AWV03-Re, No template control-Re, No template control-2 (The suffix “-Re” indicates the PCR product of the nested PCR).


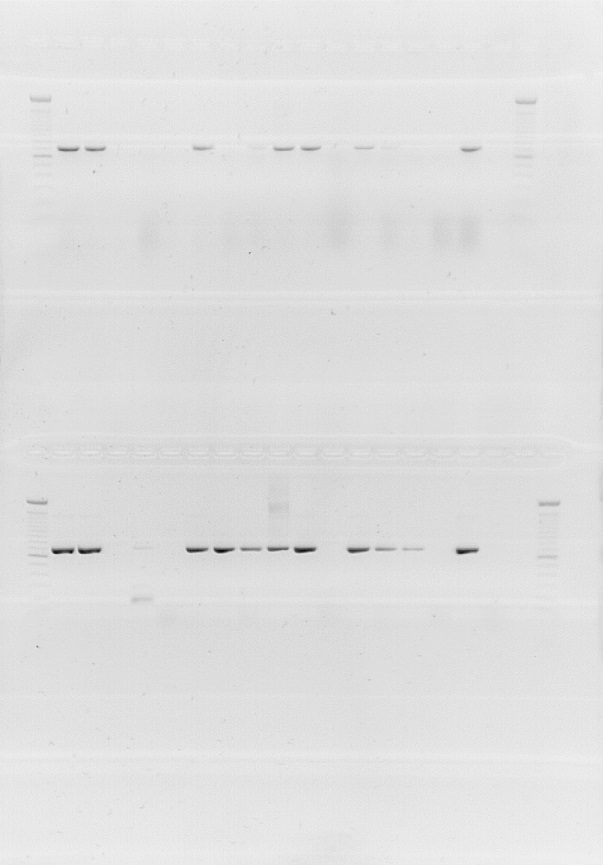


**Supplementary Figure 4**. Gel Electrophoresis of the products of amplification of mitochondrial cytochrome c oxidase subunit I from DNA extracts of armyworms. In the top lane, samples were loaded in the following order: 100bp size marker, CABI-AWB04, CABI-AWB05, CABI-AWB06, CABI-AWB07, CABI-AWB08, CABI-AWB09, CABI-AWB10, CABI-AWB11, CABI-AWB12, CABI-AWB13, CABI-AWN04, CABI-AWN05, CABI-AWV04, CABI-AWV05, CABI-AWV06, CABI-AWV07, No template control, 100bp size marker. In the bottom lane, samples were loaded in the following order:: 100 bp, CABI-AWB04-Re, CABI-AWB05-Re, CABI-AWB06-Re, CABI-AWB07-Re, CABI-AWB08-Re, CABI-AWB09-Re, CABI-AWB10-Re, CABI-AWB11-Re, CABI-AWB12-Re, CABI-AWB13-Re, CABI-AWN04-Re, CABI-AWN05-Re, CABI-AWV04-Re, CABI-AWV05-Re, CABI-AWV06-Re, CABI-AWV07, No template control-Re, No template control-2, 100bp size marker (The suffix “-Re” indicates the PCR product of the nested PCR).
